# The relationship between maternal dietary patterns during pregnancy in women with gestational diabetes mellitus and infant appetitive feeding behaviour at 6 months

**DOI:** 10.1038/s41598-020-77388-1

**Published:** 2020-11-25

**Authors:** Emma Amissah, Gregory D. Gamble, Clare R. Wall, Caroline A. Crowther, Jane E. Harding

**Affiliations:** 1 Liggins Institute, University of Auckland, 85 Park Rd, Grafton, Auckland, 1023 New Zealand; 2 Centre for Longitudinal Research—He Ara ki Mua, University of Auckland, Auckland, 1072 New Zealand; 3 Discipline of Nutrition and Dietetics, School of Medical Sciences, University of Auckland, Auckland, 1072 New Zealand

**Keywords:** Nutrition, Paediatrics, Paediatric research

## Abstract

Early dietary exposure may influence infant appetitive feeding behaviour, and therefore their later health. Maternal diabetes in pregnancy is associated with an increased risk of obesity in the offspring. We, therefore, examined third-trimester dietary patterns of women with gestational diabetes, their offspring’s appetitive feeding behaviour at 6 months of age, and relationships between these. We used data from a prospective cohort of women with gestational diabetes and assessed maternal dietary patterns at 36 weeks’ gestation using principal component analysis; infant appetitive feeding behaviour at 6 months of age using the Baby Eating Behaviour Questionnaire; and relationships between these using general linear modelling and chi-square tests. In 325 mother-infant dyads, we identified three distinct maternal dietary patterns: ‘Junk,’ ‘Mixed,’ and ‘Health-conscious.’ The maternal ‘Health-conscious’ pattern was inversely associated with ‘enjoyment of food’ in their sons (β − 0.24, 95% CI − 0.36 to − 0.11, *p* = 0.0003), but not daughters (β − 0.02, 95% CI − 0.12 to 0.08, *p* = 0.70), and was positively associated with ‘slowness in eating,’ (β 0.13, 95% CI 0.02 to 0.24, *p* = 0.01). Third-trimester dietary patterns in women with gestational diabetes may have sex-specific effects on infant appetitive feeding behaviour at 6 months of age.

## Introduction

A healthy appetite is important for an infant’s optimal growth and long-term health. Appetite is defined as “the internal driving force for search, choice, and ingestion of food”^1^, and growing evidence suggests that maternal diet in the prenatal and early postnatal period may alter an infants’ appetitive feeding behaviour via a panoply of hormones, genes, and mechanisms including orosensory controls^2^. Flavour, a major determinant of food preference, is essential in the development of infant appetitive feeding behaviour^3,4^. It is thought that the developing fetus is first exposed to flavour in utero via maternal dietary components in the amniotic fluid^5–7^ and that the initial exposure may mould future dietary proclivities. For instance, infants of mothers who ate a variety of foods during pregnancy and breastfeeding are reported to be more tolerant of a wide array of flavours compared to their formula-fed counterparts^8^. Similarly, a cohort study in the UK has reported significant associations between maternal protein and fat intake at 32 weeks of gestation and the offspring’s protein and fat intake at 10 years of age^9^.

Animal studies support these findings and have shown that alterations in the nutritional environment during gestation may program appetite and the feeding behaviour of the offspring^10–13^ Appetite is thought to be regulated by neurobiological processes involving the homeostatic and hedonic systems^14^. While the homeostatic system boosts eating to satisfy energy needs, hedonic systems are mediated by the associated reward, e.g., palatability of the food, which can encourage eating even beyond energy needs^15^. The hypothalamus is critical to the optimal functioning of these systems, and alterations in the nutritional environment during critical periods of growth may alter the development of the hypothalamus with a risk for later adverse health consequences^16^. For example, in rats, high-fat feeding from pre-conception through lactation alters hypothalamic gene expression in the offspring, which may lead to abnormal formation of neuronal projections and the neuronal circuitry controlling appetite in later life^10,17^. It is also interesting to note that in animals and humans maternal diet in pregnancy, e.g., high fat diet has been shown to play a role in the early colonization of the offspring’s gut^18,19^. The altered microbiome may then produce metabolites which may epigenetically modify key genes involved in the regulation of the offspring’s appetite^20–23^.

Infant appetitive feeding behaviour includes traits such as food responsiveness, slowness in eating, and satiety responsiveness, which contribute to variations in adiposity and weight gain in infants^24,25^. For instance, food responsiveness measured at 3 months of age is significantly associated with higher BMI z-scores from 6 to 15 months of age and higher weight gain between 3 and 6 months of age^26^. In contrast, slowness in eating and satiety responsiveness is significantly associated with lower BMI z-scores at 6 months and with less weight gain between 3 and 6 months of age^27^. Associations between adiposity, satiety responsiveness, and food responsiveness have also been reported among groups of children aged between 3 to 5 and 8 to 11 years^28^.

Although there is some evidence that maternal diet affects infant appetitive feeding behaviour, the effects are inconsistent, and it is unclear when abnormalities in appetitive traits begin to manifest and to what extent they persist^2^. Women with gestational diabetes mellitus (GDM) are commonly overweight, at increased risk of developing type 2 diabetes and hypertensive disorders, and have large babies who are also at a higher risk of obesity and metabolic disease in later life^29,30^. However, we are not aware of any studies that have examined maternal dietary patterns in women with GDM and their relationship with appetitive traits of their offspring. Thus, our study aimed to investigate dietary patterns of women with GDM in late pregnancy, appetitive feeding behaviour in their infants, and the relationship between these. We hypothesised that (1) infants of women with GDM would have obesity-related appetitive traits such as high food responsiveness and high enjoyment of food; (2) unhealthy maternal dietary patterns, high in energy-dense, nutrient-poor discretionary foods, would be associated with higher food responsiveness, enjoyment of food and higher general appetite in infants; and (3) healthy maternal dietary patterns, high in nutrient-dense foods such as fruits and vegetables, would be associated with higher satiety responsiveness and slowness in eating in infants.

## Results

### Sample characteristics

Of the 339 women with dietary data at 36 weeks’ gestation, 8 women with 10 or more dietary items missing, and 6 with implausible energy values were excluded. A final sample of 325 mothers were included in the dietary pattern analyses. Women in the cohort were similar to, i.e., fell within the 95% confidence intervals for, all women who gave birth in New Zealand in 2017 for parity and socioeconomic status, but were slightly older, more likely to be overweight or obese, had an overrepresentation of Asian and underrepresentation of Māori ethnicities, and were less likely to smoke (Table 1).

**Table 1:** Characteristics of women and infants in the study cohort, those who did and did not respond to the infant feeding questionnaires, and all New Zealand births in 2017.

Characteristic	All NZ births (n = 59,661)		Target Cohort (n = 325)		Responders n = 247		Non-responders n = 78		*P* value
Maternal	Mean or n	SD or %	Mean or n	SD or %	Mean or n	SD or %	Mean or n	SD or %	
Age at study entry (years)	30.0*		32.4	2.8	31.1	1.8	31.6	5.1	0.11
GA at study entry (weeks)	N/A		31.2	1.9	32.6	4.7	31.1	2.2	0.80
BMI at study entry (Kg/m^2^)	N/A								
Median			30.7		30.7		31.5		0.17
Interquartile range			27.3, 36.5	27.3, 35.3			26.4, 37.9		
**BMI category (Kg/m**^2^**)**									
< 18.5—underweight	1527	2.7	0	0.0	0	0.0	0	0.0	0.61
18.5– < 25—Normal	23,985	42.4	34	10.5	25	10.2	9	11.5	
25 to < 30—Overweight	16,009	28.3	106	32.8	84	34.3	22	28.2	
≥ 30—Obese	15,008	26.5	183	56.7	136	55.5	47	60.3	
**Ethnicity**									
NZ European	26,599	44.6	149.0	45.9	125	38.5	24	7.4	0.009
Maori	14,892	25.0	32.0	9.9	22	6.8	10	3.1	
Pacific	6,008	10.1	35.0	10.8	21	8.5	14	4.3	
Asian	10,602	17.7	105.0	32.3	75	23.1	30	9.2	
Other	1,549	2.6	4.0	1.2	4	1.2	0	0.0	
**Smoking status**									
Smokers	7,411	13.1	26	8.0	22	8.9	4	5.1	0.50
Unknown	71	0.1	1	0.3	1	0.3	0	0.0	
Primiparity	22,709	40.0	142	43.7	105	42.5	37	47.4	0.44
**NZDep at study entry**									
1–2—least deprived	8,785	14.8	47	15.5	35	15.4	12	16.0	0.004
3–4	9,612	16.2	46	15.2	40	17.5	6	8.0	
5–6	10,760	18.2	43	14.2	38	16.7	5	6.7	
7–8	13,198	22.3	63	20.8	49	21.5	14	18.7	
9–10—most deprived	16,894	28.5	104	34.3	66	29.0	38	50.7	
**Infant**									
Boys	30,733	51.2	158	48.6	126	51.0	32	41.0	0.12
**GA (weeks)**									
Median	39.0*		38.7		38.7		38.9		0.20
Interquartile range			38.1, 39.3		38.1, 39.3		38.0, 39.4		
Weight (g)	3410*		3,316.0	497.0	3,314.0	469.0	3,325.0	582.0	0.87

*NZ* New Zealand, *n* total number of participants, *SD* standard deviation, *N/A* not applicable, *GA* gestational age, *BMI* body mass index, *NZDep* New Zealand Deprivation index.

*Median/mean values with no measure of spread data available; Data for all New Zealand births from the Ministry of Health, New Zealand; *p* values compare responders and non-responders.

Of the 325 mothers with dietary data, 247 (76%) completed the infant appetitive feeding behaviour questionnaire. Those who completed the questionnaire were more likely to be of Asian and New Zealand European ethnicities, and to be in the lower socioeconomically deprived quintiles compared with those who did not (Table 1).

At 6 months of age, 35 (14.2%) infants were exclusively breastfed, 152 (61.5%) were predominantly/partially breastfed, while 60 (24.3%) were exclusively formula-fed. The mean age at start of solids was 5.1 (SD 0.72) months.

Overall, ‘enjoyment of food’ for infants in this cohort was negatively skewed. The majority of infants (92.3%) were reported to have high (scores > 3.66 to 5) enjoyment of their milk and feeding times, 54.5% had a high general appetite, and 4.9% had high food responsiveness. Infants who had higher scores for ‘food responsiveness’ had higher general appetite (r = 0.44, *p* < 0.0001), and higher scores for ‘slowness in eating’ (r = 0.21, *p* < 0.0008). In contrast, infants who had higher scores for ‘enjoyment of food’ had lower scores for ‘satiety responsiveness’ (r =  − 0.22, *p* < 0.0006) and ‘slowness in eating’ (r =  − 0.22, *p* < 0.0006) (Table 2).

**Table 2:** Infant appetitive trait scores and relationships between them.

Appetitive trait				Food response		Enjoyment of food		Slowness in eating		Satiety response		General appetite
	n	Scores	SD/IQR	r	*p* values	r	*p* values	r	*p* values	r	*p* values	r
**Overall**												
Food responsiveness	247	2.37	0.75	1								
Enjoyment of food*	247	4.50**	(4.25–4.75)	− 0.04	0.56	1						
Slowness in eating	247	2.42	0.77	0.21	0.0008	− 0.22	0.0006	1				
Satiety responsiveness	247	2.42	0.71	− 0.10	0.11	− 0.22	0.0006	0.08	0.23	1		
General appetite*	246	4.00	(3.00–5.00)	0.44	< 0.0001	0.26	0.0002	− 0.06	0.57	− 0.24	< 0.0001	1
**Boys**												
Food responsiveness	126	2.42	0.74	1								
Enjoyment of food*	126	4.00**	(4.00–4.75)	0.06	0.49	1						
Slowness in eating	126	2.44	0.76	0.23	0.01	− 0.17	0.05	1				
Satiety responsiveness	126	2.39	0.71	− 0.01	0.91	− 0.21	0.02	0.15	0.10	1		
General appetite*	126	4.00**	(3.00–5.00)	0.39	< 0.0001	0.30	0.0007	− 0.07	0.42	− 0.10	0.28	1
**Girls**												
Food responsiveness	121	2.32	0.75	1								
Enjoyment of food*	121	4.50**	(4.25–4.75)	0.04	0.68	1						
Slowness in eating	121	2.40	0.78	0.19	0.03	− 0.19	0.04	1				
Satiety responsiveness	121	2.46	0.71	− 0.20	0.03	− 0.22	0.02	0.01	0.92	1		
General appetite*	121	4.00**	(3.00–4.50)	0.50	< 0.0001	0.19	0.04	− 0.01	0.95	− 0.41	< 0.0001	1

*n* number of study participants; Scores are mean or median, *SD* standard deviation, *IQR* interquartile range, *r* Pearson correlation coefficients.

*r values are Spearman correlation coefficients.

**Median values; Differences between boys and girls were tested via Wilcoxon Mann Whitney test; Missing: boys n = 32, girls n = 46.

Boys and girls had similar appetitive mean scores and showed a fair to moderate correlation between general appetite, food responsiveness to cues of feeding, and enjoyment of milk and feeding time. In girls but not boys, there was a negative relationship between satiety responsiveness and both food responsiveness and general appetite (Table 2).

### Maternal dietary pattern analysis

**Figure 1:**
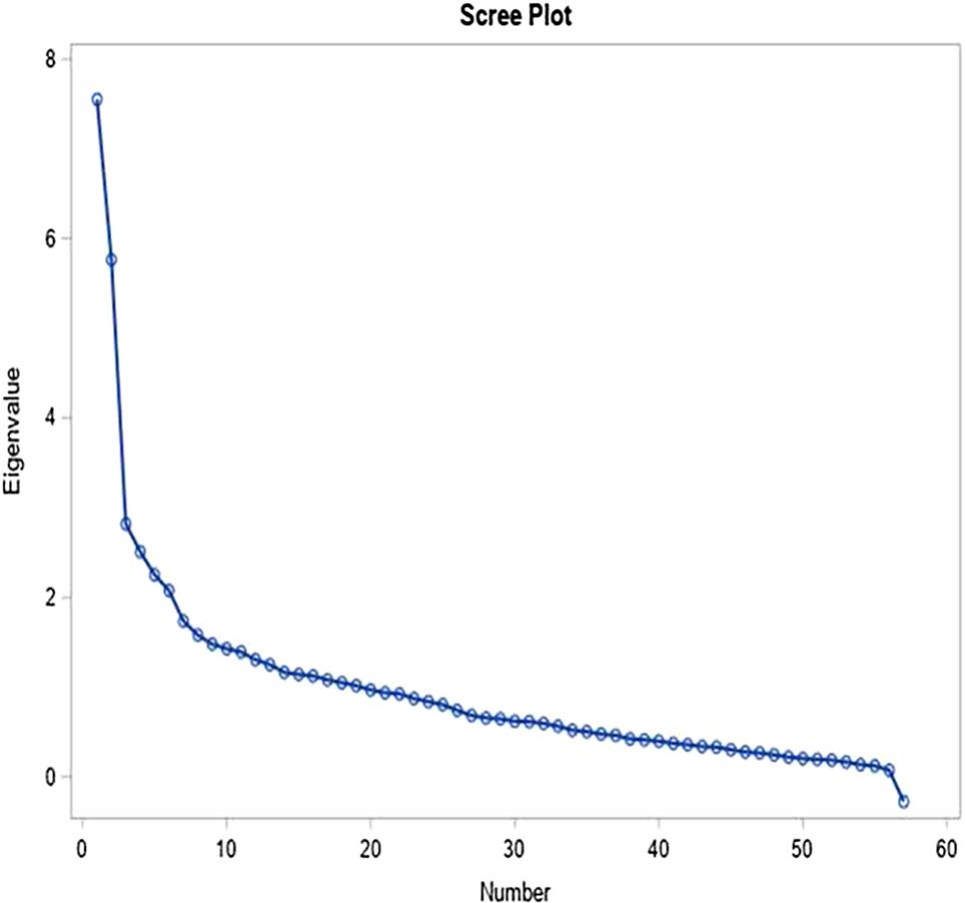
Scree plot for principal component analysis of maternal diet.

**Figure 2:**
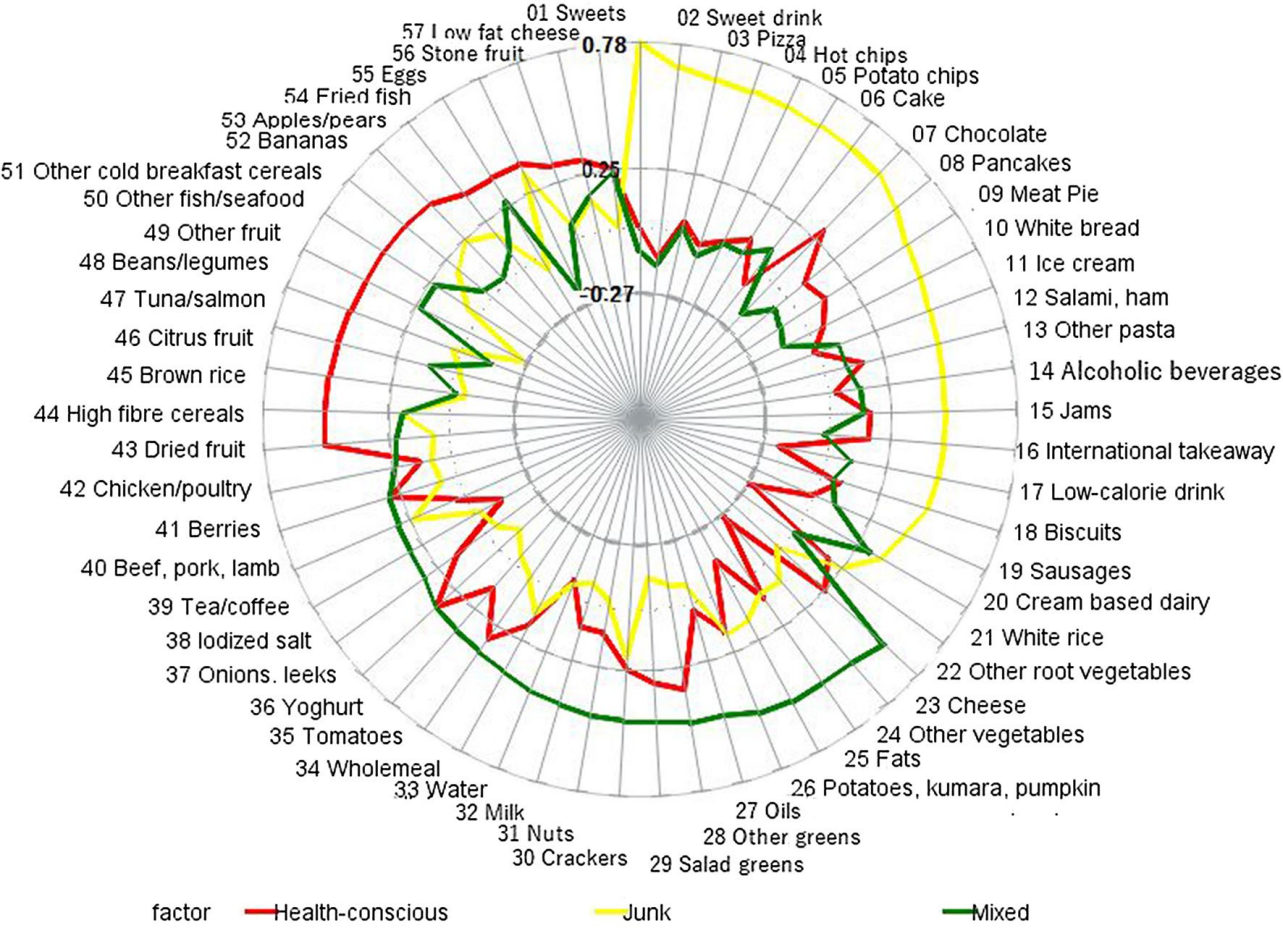
Food loadings for the three maternal dietary patterns identified.

Using the 57 food items in the semi-quantitative FFQ (food frequency questionnaire) (supplementary Table S1), the principal component analysis (PCA) showed a correlation matrix with correlation coefficients mostly below *r* < 0.6, a KMO of 0.75, and Bartlett’s sphericity test of < 0.0001. We obtained 19 components with eigenvalues greater than 1 but retained three components based on the inflection point in the scree plot (Fig. 1), and the ease of interpretability of the components (Table 3). The three components explained 28.3% of the total variation in food intake and were labelled: component 1 ‘Junk’—loaded heavily on sweets, sweet drink, pizza, hot chips, potato chips, cake, chocolate, pancake, meat pie, white bread, ice cream, salami, other pasta, alcoholic beverages, jams, international takeaway, low caloric drink, biscuit, sausages, and cream based dairy; component 2 ‘Mixed’ loaded on other root vegetables, cheese, other vegetables, fats, potatoes, kumara, pumpkin, other greens, salad greens, oils, crackers, nuts, water, tomatoes, wholemeal, yoghurt, onions, iodized salt, tea/coffee, beef/pork/lamb, chicken/poultry, and dried fruit; and component 3 ‘Health-conscious’—loaded on high fibre cereals, brown rice, citrus fruits, tuna/salmon, beans/legumes, other fruits, other fish/seafood, other cold breakfast cereals, bananas, apple/pears, fried fish, eggs, and low-fat cheese (Fig. 2). Cronbach’s coefficient *α* values (95% CI) showed good internal consistency: Junk 0.63 (0.57, 0.69); Mixed 0.81 (0.79, 0.84) and Health-conscious 0.75 (0.71, 0.79). Cronbach’s coefficient α values did not improve after removing cream-based dairy and alcoholic beverages in the Junk pattern (0.63–0.64), and tea/coffee in the mixed pattern (0.81–0.81).

**Table 3:** Principal component loadings with 57 food items and varimax rotation (n = 325).

Food items	Junk	Mixed	Health-conscious
Eigen values	7.55	5.76	2.82
Variance explained (%)	13.25	10.11	4.95
Cronbach’s alpha	0.63	0.81	0.75
Sweets	*0.7784*	− 0.09413	0.00093
Sweet drink	*0.68524*	− 0.14676	− 0.11837
Pizza	*0.65881*	0.0264	0.04888
Hot chips	*0.65467*	− 0.07471	− 0.02918
Potato chips	*0.65086*	0.01468	0.02382
Cake	*0.64452*	0.02012	0.08998
Chocolate	*0.64194*	0.10337	− 0.0833
Pancakes	*0.63916*	− 0.18644	*0.30673*
Meat pie	*0.59333*	− 0.06893	0.09492
White bread	*0.54007*	− 0.09515	0.12878
Ice cream	*0.52037*	− 0.12787	0.06492
Salami, ham	*0.49601*	0.09004	− 0.0138
Other pasta	*0.48815*	0.09588	0.16456
Alcoholic beverages	*0.48437*	0.13567	0.02714
Jams	*0.48428*	0.14241	0.16824
International takeaway	*0.47992*	− 0.02581	0.16609
Low-calorie drink	*0.47251*	0.10498	− 0.20699
Biscuits	*0.46423*	0.04862	0.08219
Sausages	*0.4092*	0.09758	− 0.01296
Cream based dairy	*0.36209*	*0.31882*	− 0.27366
White rice	0.24776	0.00451	0.17852
Other root vegetables	− 0.02101	*0.59345*	0.25889
Cheese	0.09771	*0.55337*	− 0.27023
Other vegetables	0.08724	*0.54455*	0.11415
Fats	0.15931	*0.54185*	− 0.13184
Potatoes, Kumara, Pumpkin	0.17126	*0.52506*	0.16414
Oils	− 0.0822	*0.48877*	0.02815
Other greens	− 0.09862	*0.48502*	*0.35108*
Salad greens	− 0.13754	*0.46863*	*0.30837*
Crackers	0.19862	*0.46373*	0.24556
Nuts	− 0.03038	*0.45836*	0.10968
Milk frequency	− 0.08818	*0.43764*	0.10419
Water	− 0.05373	*0.42659*	− 0.07426
Wholemeal	0.12226	*0.39859*	0.17653
Tomatoes	0.00763	*0.37979*	*0.318*
Yoghurt	− 0.04549	*0.36839*	0.13624
Onions, leeks	− 0.12324	*0.36068*	*0.35645*
Iodized salt	− 0.05264	*0.31845*	0.1389
Tea/coffee	− 0.028	*0.30626*	− 0.13268
Beef, pork or lamb	0.23454	*0.30177*	0.03514
Berries	0.06803	0.29959	0.29684
Chicken/poultry	0.08479	0.24285	0.13748
Dried fruit	0.05971	0.22141	*0.52613*
High fibre cereals	0.19444	0.18775	*0.51751*
Brown rice	− 0.06661	− 0.02483	*0.51185*
Citrus fruit	− 0.02563	0.10971	*0.50367*
Tuna/salmon	0.03411	− 0.13517	*0.4943*
Beans/legumes	− 0.24829	0.23166	*0.48325*
Other fruit	0.05913	0.226	*0.47951*
Other fish/seafood	0.18357	0.04758	*0.47213*
Other cold breakfast cereals	0.24169	0.02485	*0.4541*
Bananas	0.18327	0.08954	*0.39626*
Apples/pears	− 0.06404	0.2736	*0.38691*
Fried fish	*0.34111*	− 0.20234	*0.3848*
Eggs	0.04857	0.07098	*0.32476*
Stone fruit	0.14093	0.16122	*0.31243*
Low fat cheese	0.01441	0.24178	0.26376

Loadings ≥ 0.3 in italics.

### Associations between maternal dietary patterns and infant appetitive traits

In the unadjusted analyses, for each standard deviation increase in the maternal ‘Health-conscious’ dietary pattern, there was a decrease of 0.09 in the infant ‘enjoyment of food’ score. In multivariable regression analyses using the parsimonious model and adjusting for sex, weight-for-age z score at 6 months and NZDep (New Zealand Deprivation index) at study entry, increasing scores in the ‘Health-conscious’ maternal dietary pattern was associated with decreased scores on ‘enjoyment of food’ in boys (β − 0.24, 95% CI − 0.36 to − 0.11, *p* = 0.0003) but not in girls (β − 0.02, 95% CI − 0.12 to 0.08, *p* = 0.70, *p* = 0.004 for interaction). Independent and positive associations were also noted between the ‘Health-conscious’ pattern and ‘slowness in eating’ (β 0.13, 95% CI 0.016 to 0.24; *p* = 0.025). No other associations approached statistical significance (Table 4).

**Table 4:** Associations between maternal dietary patterns and infant appetitive traits.

Appetitive trait	n	Junk							Mixed							Health-Conscious						
		Overall			Boys		Girls		Overall			Boys		Girls		Overall			Boys		Girls	
		β	95% CI	*p* value Junk*sex	β	95% CI	β	95% CI	β	95% CI	*p *value Mixed*sex	β	95% CI	β	95% CI	β	95% CI	*p *value Health-conscious*sex	β	95% CI	β	95% CI
**Unadjusted analyses**																						
Food responsiveness	247	0.06	− 0.11, 0.22		0.10	− 0.13, 0.33	0.009	− 0.23, 0.25	0.02	− 0.08, 0.12		− 0.01	− 0.16, 0.13	0.04	− 0.10, 0.18	0.08	− 0.02, 0.19		0.05	− 0.10, 0.21	0.11	− 0.03, 0.25
Enjoyment of food	247	− 0.02	− 0.14, 0.10		− 0.09	− 0.28, 0.11	0.05	− 0.11, 0.20	0.02	− 0.05, 0.10		0.03	− 0.10,0.15	0.03	− 0.07, 0.12	− **0.09**	− **0.17, **− **0.01**		− **0.19**	− **0.31, **− **0.07**	< 0.01	− 0.09, 0.10
Slowness in eating	247	0.006	− 0.16, 0.17		0.01	− 0.22, 0.25	− 0.001	− 0.25, 0.24	0.02	− 0.08, 0.13		0.01	− 0.14, 0.16	0.03	− 0.12, 0.18	0.06	− 0.04, 0.17		0.06	− 0.09, 0.22	0.07	− 0.08, 0.22
Satiety responsiveness	247	0.02	− 0.14, 0.17		− 0.01	− 0.23, 0.20	0.05	− 0.18, 0.27	0.01	− 0.09, 0.11		− 0.05	− 0.19, 0.09	0.07	− 0.07, 0.20	0.02	− 0.08, 0.11		− 0.04	− 0.18, 0.11	0.06	− 0.08, 0.20
General appetite	246	− 0.11	− 0.36, 0.13		− 0.09	− 0.41, 0.24	− 0.14	− 0.51, 0.22	0.05	− 0.10, 0.20		− 0.06	− 0.27, 0.15	0.14	− 0.08, 0.36	− 0.09	− 0.25, 0.06		− 0.04	− 0.26, 0.18	− 0.14	− 0.37, 0.09
**Adjusted analyses**																						
Food responsiveness^a^	216	0.004	− 0.17, 0.17	0.45	0.07	− 0.16, 0.31	− 0.08	− 0.32, 0.17	0.03	− 0.07, 0.13	0.70	− 0.005	− 0.15, 0.14	0.05	− 0.09, 0.19	0.08	− 0.03, 0.18	0.57	0.05	− 0.11, 0.20	0.11	− 0.03, 0.25
Enjoyment of food^b^	216	0.05	− 0.14, 0.23	0.37	− 0.05	− 0.35, 0.25	0.09	− 0.13, 0.31	0.06	− 0.02, 0.15	0.80	0.07	− 0.07, 0.20	0.06	− 0.05, 0.17	− 0.01	− 0.12, 0.10	** < 0.01**	− **0.24**	− **0.36, **− **0.11**	− 0.02	− 0.12, 0.08
Slowness in eating^c^	216	0.03	− 0.14, 0.19	0.65	0.04	− 0.20, 0.27	0.02	− 0.23,0.26	0.01	− 0.10, 0.11	0.44	− 0.01	− 0.16, 0.14	0.02	− 0.13, 0.17	**0.13**	**0.02, 0.24**	0.85	0.12	− 0.04, 0.28	0.13	− 0.03, 0.29
Satiety responsiveness^d^	216	0.01	− 0.14, 0.16	0.84	− 0.02	− 0.23, 0.20	0.04	− 0.18, 0.26	0.01	− 0.09, 0.10	0.16	− 0.07	− 0.21, 0.08	0.08	− 0.06, 0.21	0.008	− 0.09, 0.11	0.21	− 0.06	− 0.20, 0.09	0.06	− 0.07, 0.20
General appetite^e^	216	− 0.19	− 0.44, 0.07	0.89	− 0.16	− 0.51, 0.19	− 0.20	− 0.58, 0.19	0.08	− 0.07, 0.23	0.23	− 0.02	− 0.24, 0.21	0.16	− 0.06, 0.37	− 0.07	− 0.23, 0.09	0.88	− 0.13	− 0.35, 0.08	− 0.13	− 0.36, 0.10

Significant values (*p* < 0.05) shown in bold.

*β* unstandardized regression coefficients, *CI* Confidence interval.

^a^Adjusted for ethnicity.

^b^Adjusted for maternal NZ Deprivation Index, and weight z score at 6 months of age.

^c^Adjusted for primiparity and ethnicity.

^d^Adjusted for infant gestational age at delivery.

^e^Adjusted for weight-for-age z score at 6-months, age at start of solids, and whether predominantly breastfed or not at 6 months of age.

## Discussion

In a cohort of 325 women with GDM in New Zealand, we identified three distinct maternal dietary patterns, labelled as ‘Junk,’ ‘Mixed,’ and ‘Health-conscious’, that explained the most variation in food intake assessed at 36 weeks’ gestation. We found, as hypothesised, that the majority of infants in our cohort had a high enjoyment of food score, although fewer than 5% had high food responsiveness. Additionally, we found that overall, the ‘health-conscious’ maternal dietary pattern was inversely associated with ‘enjoyment of food’ in boys but not in girls, and positively associated with ‘slowness in eating’ at 6 months of age.

Several animal studies have examined the effect of maternal diet on feeding behaviour^31,32^ and neurobiological processes^33–35^ controlling appetite in the offspring. However, to the best of our knowledge, this is the first study to assess the effect of third-trimester diet in women with GDM on a questionnaire-based assessment, i.e., the Baby Eating Behaviour questionnaire (BEBQ) of infant appetitive feeding behaviour at 6 months of age. Our results suggest that a ‘Health-conscious’ maternal dietary pattern is associated with better appetitive control in infants at 6 months of age. Appetitive traits influence an infant’s weight gain^36^. In particular, while enjoyment of food is positively associated with a higher risk of overweight in childhood^37,38^, slowness in eating is negatively associated^38^. Therefore, our findings suggest that a ‘Health-conscious’ dietary pattern in women with GDM may potentially decrease the risk of later overweight/obesity of their offspring, particularly in boys^39,40^.

The reason for these sex-specific associations is unclear. Sex differences are reported in the prevalence of obesity, with boys at a higher risk across all age groups^41^. Additionally, it has been reported in some studies that children eat fewer fruits, vegetables, and whole grains than recommended^42^, and boys are reported to have higher preferences for meat, fish, poultry, and high-fat foods compared to girls who have a higher liking for fruits and vegetables^43–45^. It would be interesting, therefore, to determine whether the maternal health-conscious dietary pattern predicts later dietary preferences, and particularly in boys.

Our findings provide new information about the dietary patterns of women with GDM in late pregnancy. Diet is the cornerstone in the management of women with GDM, yet few studies^46,47^ have examined maternal dietary patterns in these women. A cohort study conducted in South Korea assessed dietary patterns via factor analysis using a 3-day food record, although the timing of dietary data collection was unclear^47^. Among the 166 women with GDM two dietary patterns were identified: a “carbohydrate and vegetable pattern” with high loadings of fruits, rice and cereals, fermented vegetables, vegetables, and meat and a “western pattern” characterized by poultry and eggs, fast food, deep-fried food, processed meat and seafood, snacks and desserts, coffee and other beverages, and seaweeds^47^. While there are similarities between the ‘carbohydrate and vegetable pattern’ and ‘western pattern’ and our ‘Mixed’ and ‘Junk’ patterns, respectively, some diversity is to be expected from the differences across cultures and methodologies. A recent longitudinal cohort study assessed differences in dietary patterns between 280 women with GDM and 5104 without GDM in New Zealand from already established dietary patterns among the cohort^46,48^. The authors reported four dietary patterns via PCA and using FFQ in the third trimester of pregnancy: ‘Junk,’ ‘Traditional/White bread,’ ‘Fusion Protein’ and ‘Health conscious’^46,48^. Their dietary patterns were comparable to the patterns defined in our study, supporting generalisability of the patterns among women with GDM in New Zealand.

The three dietary patterns identified in our study explained only 23.8% of the variation in food intake, which could be interpreted as indicating insufficient summarising of the dietary data or possibly that other unidentified phenomena may be present. However, while the regrouping of a large number of different food items, e.g., apples, pears, etc. from the FFQ into a single fruit group markedly improved the percentage of variability explained in our analyses, there was an inevitable trade-off in loss of detail. Thus, we chose not to regroup to reduce the food items as greater detail is reported to improve the precision by which dietary patterns estimate disease risk^49^. Additionally, the total percent of variance explained by the dietary patterns in our study is consistent with similar studies conducted among pregnant^50,51^ and non-pregnant women^52^ in New Zealand, where the percentage of variance explained was 13.8–25%.

This study has several strengths. Firstly, we used a standardised and validated psychometric measure, the BEBQ, to quantify appetitive traits in the infants and a FFQ that was adapted to be culturally appropriate for New Zealand. Secondly, the dietary patterns identified showed good internal consistency, which supports the reliability of the Likert-type scales used in the FFQ. Thirdly, we employed a prospective study design and began follow-up of the infants early postpartum, which will permit further analyses of changes in appetitive behaviour into adulthood in the future.

Women in our study cohort may not be representative of the GDM population in New Zealand, as responders differed from non-responders in ethnicity and socioeconomic status. Other limitations lie in methodologies used in collecting and analysing the maternal dietary and infant appetitive feeding behaviour data. Although the conduct and analysis of food diaries in large trials such as TARGET are deemed cost-prohibitive and time consuming compared to FFQs, food diaries involve real-time documentation of food consumption. Our sole reliance on maternal self-report of dietary intake and infant feeding behaviour, as opposed to observed measures of data collection, may have introduced recall and social desirability bias^53–55^. However, the FFQ we used was shown to have acceptable to good validity when compared to the 8-day dietary record^56^, and dietary patterns derived from PCA and food records are reported to be similar^57–59^. Additionally, PCA is inherently subjective, with a tendency towards interpretation bias^60,61^. However, given the similarities between our study dietary patterns and a prior study^46^, the likelihood of interpretation bias in this study appears low. Also, although we adjusted extensively for potential confounding factors, we cannot rule out the possibility of residual confounding.

This exploratory study provides new insights into the relationships between maternal dietary patterns in women with GDM, and the possible impact of maternal diet during pregnancy on the appetitive feeding behaviour of their infants. Although the ‘Health-conscious’ maternal dietary pattern was related to appetitive feeding behaviours in boys that might be expected to result in decreased obesity risk, it is not known whether these relationships persist, or have any relationship with later growth. Further assessment of these infants as they grow older, are introduced to diverse foods, and transition to independent feeding may help to ascertain long-term health implications.

## Conclusion

Appetitive control in infants at high risk of later obesity may be affected by maternal dietary patterns during late pregnancy. We found evidence that among women with GDM, the ‘Health-conscious’ maternal dietary pattern in the third trimester of pregnancy is associated with better appetitive control, particularly in boys, at 6 months of age. Since the risk of obesity is increased in the offspring of women with GDM, appropriate dietary advice in pregnancy may be a potential target for intervention and public health recommendations.

## Methods

### Study design and setting

This nested cohort study is a secondary analysis of data from the TARGET Trial, a multicentre, stepped-wedge, randomised trial that compared the effects of tighter treatment targets for glycaemic control in women with GDM with less tight targets on maternal and infant outcomes^62^. Details of the trial protocol are described elsewhere^62^. Briefly, women with a singleton pregnancy and diagnosed with GDM by an oral glucose tolerance test ≥ 22 weeks’ gestation were recruited from 10 hospitals in New Zealand between 2015 and 2017. The TARGET trial was approved by the Northern A Health and Disability Ethics Committee (14/NTA/163/AMO1). Participants gave written informed consent. TARGET was registered with the Australian New Zealand Clinical Trials Registry—ACTRN 12615000282583. No additional consent was required for the current study using anonymised data from the TARGET Trial, but the study was carried out in accordance with the principles of the Declaration of Helsinki and the University of Auckland Research Code of Conduct.

### Participants

Women with GDM who were recruited to the TARGET Trial were included in this nested study with their infants if they had completed a food frequency questionnaire at 36 weeks’ gestation about their diet during the trial; had data on infant sex, gestational age at birth, and pregnancy outcomes; and had participated in the follow-up at 6-months after birth.

### Outcomes and measures

#### Maternal diet

Data on food consumption was collected from study participants via a self-administered, 1-month recall, FFQ at 36 weeks’ gestation. The FFQ was a customized version of the 163-item semi-quantitative FFQ developed by Willett^63^, with changes made to reflect local dietary habits as per the nationally-representative nutrition survey available at the time^64^, and further informed by two focus groups of 21 adults aged 30–59 years in New Zealand^56^. The questionnaire comprised 65 questions, of which 57 were grouped as dairy, eggs and meat, fish and seafood, bread, cereals and starches, fruits, vegetables, fast foods, beverages, sweets, baked goods, and miscellaneous (Supplementary Table S1). Food frequencies were measured on a six-point ordinal scale ranging from “never or less than once per month” to “4–6 times per day”^56^.

We excluded data from women with more than ten dietary items missing and assumed an item was never consumed for missing data where ten or fewer items were missing^65^. We also excluded implausible data with reported energy intakes < 500 and > 3500 kcal/day^66^.

#### Dietary patterns

Of the 65 food questions in the semi-quantitative FFQ, we used 57 to conduct the PCA, excluding items which were intended for cross-validation and items that were not food group based (Supplementary Table S1). We converted the ordinal maternal dietary data to weekly frequencies of food consumption as follows: never or less than a month = 0, 1–3/month = 0.5, 1/week = 1, 2–4/week = 3, 5–6/week = 5.5, 1/day = 7.0, 2–3/day = 17.5, 4–6/day = 35. To obtain a smaller set of variables that explained maximum variations in the dietary patterns of women with GDM, we performed PCA using the polychoric correlation matrix, which is more appropriate for ordinal data from Likert-type rating scales, and varimax rotation^67^. We used the PROC FACTOR, method = principal statement in SAS^68^, and assessed the suitability of the maternal dietary data for PCA via the correlation matrix, Kaiser–Meyer–Olkin (KMO) measure of sampling adequacy, and Bartlett’s sphericity test. KMO values range between 0 and 1, and values closer to 1 affirm the suitability of the data for PCA, while values < 0.50 are unacceptable^69,70^. We considered Bartlett’s *p* value less than 0.05 as acceptable and indicating a significant difference between the observed correlation matrix and the identity matrix^71^. We also assessed the reliability of the Likert-type scales used in the FFQ by evaluating the internal consistency of food items in each identified dietary pattern via Cronbach’s coefficient alpha (*α*). We considered Cronbach's coefficient *α* values ≥ 0.70 as good^72^ and removed food items with poor item-total correlations while assessing the effect of the removal on the reliability of the scale^60^. We used eigenvalues > 1, the breakpoint in the scree plot, and the interpretability of the components after varimax rotation to determine the number of components to retain^73–75^. We used food items with absolute loading values of ≥ 0.3 to characterise the identified dietary patterns. We calculated weighted component scores as standardised variables and divided subjects into tertiles based on their scores for further analyses. Higher factor scores indicated the degree to which a woman’s diet adhered to the identified dietary pattern^72^.

#### Infant feeding practice

Data on infant breastfeeding status and age of introduction of solid food were collected from mothers at 6 months postpartum by questionnaire. We defined exclusively breastfed infants as having received only breast milk and no other liquids or solid foods, except for prescribed medicines, and predominantly breastfed infants as having received breast milk together with other liquids^76,77^.

#### Infant feeding behaviour

Data were collected from mothers at 6-months postpartum via a self-administered infant feeding behaviour questionnaire, based on the BEBQ^78^. The BEBQ was adapted from the validated Children’s Eating Behaviour Questionnaire and modified for use in infants during the exclusive milk-feeding stage^79^. The BEBQ consists of 18 items developed to measure general appetite and four specific appetitive traits: ‘enjoyment of food’ (4 items), ‘food responsiveness’ (6 items), ‘slowness in eating’ (4 items), and ‘satiety responsiveness’ (3 items) (Supplementary Table S2). We measured general appetite with the question, ‘my baby had a big appetite’ and recorded maternal responses on a 5 point Likert frequency scale with response options of: ‘never,’ ‘rarely,’ ‘sometimes,’ ‘often,’ and ‘always.’ We reverse-scored two items, i.e., “my baby became distressed while feeding” and “my baby finished feeding quickly,” calculated mean scores for each appetitive trait, and grouped appetitive scores as low (1 to ≤ 2.33), medium (> 2.33 to ≤ 3.66), and high (> 3.66 to 5)^80^, with high scores indicating higher traits.

#### Covariates

We considered the following covariates for each appetitive variable: continuous—maternal age and total daily energy intake, infant gestational age at birth, birth weight, weight and weight-for-length z scores at 6 months and age at introduction of solids; categorical—prioritised ethnicity (European; Māori; Pacific Peoples; Asian; and Other), and BMI (underweight < 18.5 kg/m^2^, normal weight 18.5–24.9 kg/m^2^, overweight ≥ 25 − 29.9 kg/m^2^, and obese ≥ 30 kg/m^2^)^81^; smoking (current smokers vs. non-smokers), parity (primiparous vs. multiparous),TARGET Trial treatment group (tighter vs. less tight glycaemic control), and infant breastfeeding status (exclusively breastfed, predominantly breastfed or formula-fed up to 6 months).

Variable selection was done on the basis of parsimony and biological plausibility after inspection of models fitted to each appetitive variable using a stepwise, Max R square, forward and backward selection. .

NZDep reflects average degrees of socioeconomic deprivation at the mesh block level, using nine variables (income, employment, communication, transport, support, qualification, owned home, and living space) combined from the 2013 census to reflect eight dimensions of deprivation^82,83^. We divided the NZDep into quintiles with the first quintile representing the least deprived 20%, and the fifth quintile the most deprived 20%. We calculated total daily energy intake from the maternal FFQ data, while all other maternal covariates were assessed via trial entry questionnaires.

### Statistical analysis

We conducted the data analyses using SAS version 9.4 (SAS Institute Inc., Cary, NC, USA). We assessed cohort characteristics, compared mother–child dyads with and without infant appetitive feeding data, and maternal reports of their infant’s appetitive traits via means and standard deviations for continuous variables, median (inter-quartile range) for skewed data, and proportions with their respective percentages for categorical variables. We also assessed the normality of the data and relationships between the BEBQ subscales, using Pearson’s product-moment correlation for normal data or Spearman correlation for skewed data. We interpreted correlation coefficients > 0.80–1.00 as strong, > 0.50–0.80 as moderate, > 0.20–0.50 as fair to weak, and 0.00–0.20 as negligible^72^.

We assessed relationships between maternal dietary pattern scores at 36 weeks’ gestation (independent variable) and BEBQ subscale scores (dependent variable) at 6 months of age using general linear models and adjusting for variables found to be associated with infant appetitive traits including infant sex, gestational age, birth weight, weight-for-age, weight-for-length, z-scores at 6 months of age, and the maternal covariates previously listed^43,84,85^. In addition to the overall models, we conducted the analyses separately for infants of each sex and examined plots of residuals for evidence of normality, homoscedasticity, the constancy of variance and outliers. Where residuals of dependent variables were not normally distributed, we explored Box-Cox power transformations to improve the curvilinearity.

## Supplementary information

Supplementary Information.

## Data Availability

Published data are available to approved researchers under the data sharing arrangements provided by the Maternal and Perinatal Central Coordinating Research Hub (CCRH), based at the Liggins Institute, University of Auckland (https://wiki.auckland.ac.nz/researchhub). Metadata, along with instructions for data access, are available at the University of Auckland’s research data repository, Figshare (https://auckland.figshare.com). Data access requests are to be submitted to Data Access Committee via researchhub@auckland.ac.nz. Data will be shared with researchers who provide a methodologically sound proposal and have appropriate ethical and institutional approval. Researchers must sign and adhere to the Data Access Agreement that includes a commitment to using the data only for the specified proposal, to store data securely and to destroy or return the data after completion of the project. The CCRH reserves the right to charge a fee to cover the costs of making data available, if required.
