# Supplementary material for: The relationship between maternal dietary patterns during pregnancy in women with gestational diabetes mellitus and infant appetitive feeding behaviour at 6 months
Source: Sci Rep. 2020 Nov 25;10:20516. doi: 10.1038/s41598-020-77388-1 (PMC7689419; doi:10.1038/s41598-020-77388-1)
Supplement: Supplementary file 1 — Supplementary Information. [file 41598_2020_77388_MOESM1_ESM.pdf]

SUPPLEMENTARY INFORMATION

The relationship between maternal dietary patterns during pregnancy in women with gestational diabetes mellitus and infant appetitive feeding behaviour at six months

Authors

Emma Amissah[1], Greg Gamble[1], Clare R. Wall[2, 3], Caroline A. Crowther[1], Jane E. Harding[1*]

Institutions

[1]Liggins Institute, University of Auckland, Auckland 1023, New Zealand

[2]Centre for Longitudinal Research—He Ara ki Mua, University of Auckland, Auckland 1072, New Zealand

[3] Discipline of Nutrition and Dietetics, School of Medical Sciences, University of Auckland, Auckland 1072, New Zealand

Corresponding author contact

*Liggins Institute, University of Auckland, 85 Park Rd, Grafton, Auckland 1023, NZ

Email: j.harding@auckland.ac.nz

Tel: +64 9 9236439

Supplementary Table S1. Summary of food groupings included in the dietary analysis

Food groups	Food items (65) from FFQ	Examples of foods	Food items used in PCA (57)
	Dietary supplement last month	Dietary supplements taken over the last month	Milk frequency
	Sugar teaspoons/day	Sugar added to beverages or food	Ice cream
Dairy Foods	Milk frequency	Cow, soy milk in hot drinks, cereals, creamed soups, etc.	Yogurt
	Ice cream	Ice cream	Low-fat cheese
	Yogurt	Yogurt	Cheese
	Low-fat cheese	Cottage, ricotta, low-fat cheddar	Cream-based dairy
	Cheese	Cheddar, edam, tasty, mozzarella, brie, camembert	Bananas
	Cream-based dairy	Cream, sour cream, cream cheese	Apples/pears
Fruits	Bananas	Bananas	Citrus fruit
	Apples/pears	Apples/pears	Stone fruit
	Citrus fruit	Oranges, mandarins, grapefruit, lemons	Berries
	Stone fruit	Apricots, plums, nectarines, peaches	Dried fruit
	Berries	Fresh, frozen or canned, e.g., strawberries, blueberries	Other fruit
	Dried fruit	Raisins, sultanas, prunes	Tomatoes
	Other fruit	Kiwifruit, grapes, feijoa, pineapples, mango, rhubarb, Tamarillos, guava, pawpaw, melon	Beans/legumes
Vegetables	Tomatoes	Fresh, canned, tomato-based sauce	Salad greens
	Beans/legumes	Green beans, runner beans, baked beans, Lentils, Chickpeas	Other greens
	Salad greens	Lettuce, cucumber, celery, rocket	Onions, leeks
	Other greens	Broccoli, cauliflower, spinach, silver beet, cabbage, Brussel sprouts, bok choy, Chinese cabbage, watercress, Puha	Potatoes, kumara, pumpkin
	Onions, leeks	Onions, leeks	Other root vegetables
	Potatoes, kumara, pumpkin	Potatoes, kumara, pumpkin	Other vegetables
	Other root vegetables	Carrot, beetroot, parsnip, turnips, yams	Eggs
	Other vegetables	Corn, mushrooms, asparagus, courgette, eggplant, Capsicum, peas, coleslaw	Sausages
Eggs, meat, etc.	Eggs	Eggs	Salami, ham
	Sausages	Sausages and hotdogs,	Beef, pork or lamb
	Salami, ham	Salami, ham luncheon, bacon or other processed meat	Meat pie
	Beef, pork or lamb	Mince, roast, steak, stew, casserole, lasagne, frozen dinners, etc.	Chicken/poultry
	Meat pie	Meat pie and two sausage rolls fried fish	Tuna/salmon
	Chicken/poultry	Chicken and other poultry	Fried fish
	Tuna/salmon	Tuna, salmon, sardines, and mackerel,	Other fish/seafood
	Fried fish	Battered fish, breaded fish, fish fingers	High fibre cereals
	Other fish/seafood	Other fish and seafood	Other cold breakfast cereals
Breads, cereals, starches	High fibre Cereals	Porridge, muesli, bran flakes, all bran	White bread
	Other cold breakfast cereals	Light 'n' tasty, special k, Weetabix	Wholemeal
	White bread	Sliced, tortillas, pita, etc.	Crackers
	Wholemeal	Sliced, tortillas, pita, etc.	Pancakes
	Crackers	Crispbread, e.g., vita-wheat, crus-kits	Brown rice
	Pancakes	Pancakes, waffles, sweet buns, scones	White rice
	Brown rice	Brown rice/ wholemeal pasta	Other pasta
	White rice	White rice/couscous	Hot chips
	Other pasta	Spaghetti, Spirals, Instant noodles, Tinned	Potatoes chips
Fast foods	Hot chips	Hot chips/French fries	Pizza
	Potatoes chips	Potato Chips, Crisps or Corn Chips	International takeaway
	Pizza	Pizza/Hamburgers	Low-calorie drink
	Intl Takeaway	International Takeaway, e.g., Chinese, Thai, Japanese, Turkish, Indian, etc.	Sweet drink
Beverages	Low-calorie drink	Diet Coke, Coke, Sprite	Alcoholic beverages
	Sweet drink	Sweet drinks, e.g., Sprite, Coke, Fruit juice, Raro, Cordial	Water
	Alcoholic beverages	Alcoholic Beverages, e.g. Beer, Wine, Spirits	Tea/Coffee
	Water	Water: Bottled, Sparkling, or Tap	Chocolate
	Tea/Coffee	Tea/Coffee	Sweets
Sweets, baked good, Miscellaneous	Chocolate	Chocolate, Chocolate bars	Biscuits
	Sweets	Sweets, Lollies	Cake
	Biscuits	Biscuits	Jams
	Cake	Cake, Brownie, Slice, Croissant, Pie, Danish, Brioche, Milk Pudding, Muesli bars	Nuts
	Jams	Jams, Preserves, Syrup, Honey	Oils
	Nuts	Nuts (including peanut butter), seeds	Fats
	Oils	Oils, e.g., Vegetable oil, Olive oil, Mayonnaise, Salad dressing, include frying	Iodized salt
	Fats	Fats, e.g., butter or margarine, used as spreads or in cooking, excluding baking	
	Iodized salt	At table and in cooking	
For cross-validation	Milk type	None, Full cream or farmhouse, Standard or homogenized, Semi trim, trim, soy milk (regular/light), other milk	
	Fruit servings/day	Fruit servings/day	
	Vegetable servings/day	Vegetable servings/day	
	Breads/day	Breads/day	
	Meat servings/week	Meat servings/week	
	Fish servings/week	Fish servings/week	

Adapted from Willet [1]

Supplementary Table S2: Summary of items included in the infant feeding behaviour questionnaire

**Scale**	**Subscale**	**Definition**	**Original items**
Food approach eating behaviours	Food Responsiveness	Measures feeding in relation to how demanding the infant is and their responsiveness to environmental food cues.	• If given a chance, my baby would always be feeding<br>• Even when my baby had just eaten well, he/she was happy to feed again if offered<br>• My baby could easily take a feed within 30 minutes of the last one.<br>• My baby was always demanding a feed.<br>• If allowed to, my baby would take too much milk.<br>• My baby frequently wanted more milk than I provided.
	Enjoyment of Food	This refers to the infant's general interest in milk and feeding, i.e., the extent to which eating is pleasurable.	• My baby seemed contented while feeding.<br>• My baby enjoyed feeding time.<br>• My baby loved milk.<br>• My baby became distressed while feeding.
Food avoidance eating behaviours	Satiety Responsiveness	Measures the ease with which an infant perceives fullness.	• My baby got full up easily.<br>• My baby got full before taking all the milk I think he/she should have.<br>• My baby found it difficult to manage a complete feed.
	Slowness in Eating	Measures the speed of feeding in infants during a meal.	• My baby fed slowly<br>• My baby finished feeding quickly.<br>• My baby took more than 30 minutes to finish feeding<br>• My baby sucked more and more slowly during the course of a feed.

Adapted from the BEBQ [2] **and** [3]

References:

1. Willett, W.C.; Sampson, L.; Stampfer, M.J.; Rosner, B.; Bain, C.; Witschi, J.; Hennekens, C.H.; Speizer, F.E. Reproducibility and validity of a semiquantitative food frequency questionnaire. *Am. J. Epidemiol.* **1985**, *122*, 51–65, doi:10.1093/oxfordjournals.aje.a114086.
2. Llewellyn, C.H.; van Jaarsveld, C.H.M.; Johnson, L.; Carnell, S.; Wardle, J. Development and factor structure of the Baby Eating Behaviour Questionnaire in the Gemini birth cohort. *Appetite* **2011**, *57*, 388–396, doi:10.1016/j.appet.2011.05.324.
3. Carnell, S.; Benson, L.; Pryor, K.; Driggin, E. Appetitive traits from infancy to adolescence: using behavioral and neural measures to investigate obesity risk. *Physiol. Behav.* **2013**, *121*, 79–88, doi:10.1016/j.physbeh.2013.02.015.
